# 10. Quadrivalent M2SR (M2-deficient Single Replication) Live Influenza Vaccine Provides Better Protection Than Inactivated Vaccine Against Drifted Influenza B Virus Challenge in Ferrets

**DOI:** 10.1093/ofid/ofab466.212

**Published:** 2021-12-04

**Authors:** Lindsay Hill-Batorski, Yasuko Hatta, Michael Moser, David Marshall, Pamuk Bilsel

**Affiliations:** FluGen, Madison, Wisconsin

## Abstract

**Background:**

Quadrivalent inactivated influenza vaccines (QIV) induce neutralizing antibodies (Abs) against the viral hemagglutinin (HA). Despite annual update of HA vaccine antigens to match circulating strains, current vaccines provide ~60% vaccine effectiveness (VE). QIV VE can be as low as 10% when circulating strains do not match vaccine HA. The live M2SR (M2-deficient single replication) influenza vaccine candidate has previously shown broad humoral, mucosal and cellular immune responses and protection against multiple influenza A subtypes. Here we show similar properties with the Quadrivalent M2SR (Quad M2SR) against drifted influenza B challenge in comparison to QIV.

**Methods:**

Ferrets pre-infected with influenza H1N1 and B/Yamagata viruses, were immunized intranasally (IN) with PBS (Mock) or Quad M2SR, or intramuscularly with Fluzone QIV. Serum collected post-vaccination was evaluated for Ab responses. Forty-two days after vaccination, ferrets were challenged IN with 10^6^ pfu of B/Brisbane/60/2008 (Victoria lineage) influenza virus. Nasal washes were taken for 7 days post-challenge and evaluated for challenge virus by TCID_50_ assay. Nasal turbinates, trachea and lungs were also evaluated for virus.

**Results:**

Quad M2SR and QIV elicited high serum Abs against the vaccine strain B/Colorado/06/2017 (Fig. 1A) and against the drifted influenza B challenge strain B/Brisbane/60/2008 (Fig. 1B) in ferrets with preexisting immunity. Like Mock, ferrets who received QIV displayed both weight loss (6.2%, Fig. 2A) and a rise in temperature (1.1^o^C, Fig. 2B) after challenge. In contrast, the Quad M2SR group did not exhibit any significant weight or temperature changes after challenge. Quad M2SR controlled the drifted challenge virus better than QIV as evidenced by significantly lower or absent post-challenge virus titer in nasal washes (Fig. 3A) and nasal turbinates (Fig. 3B).

Figure 1. Serum Neutralization Titers Post-Vaccination

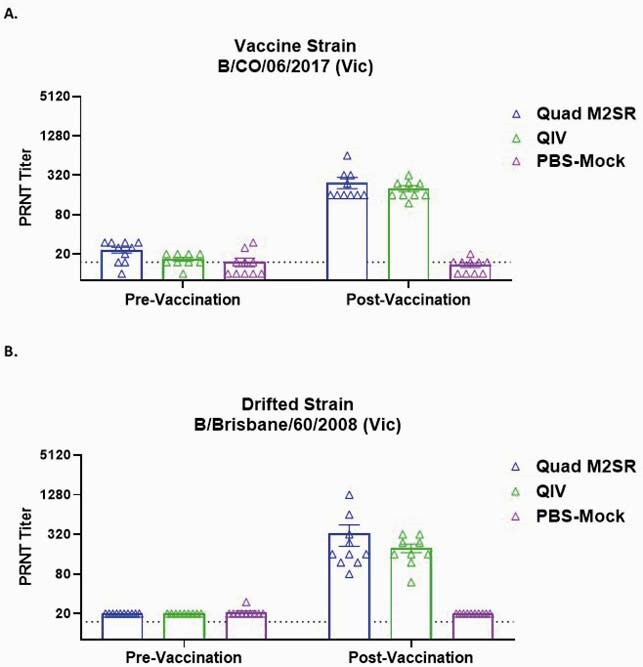

Plaque reduction neutralization test (PRNT) antibody titers for Quad M2SR and QIV against matched Influenza B vaccine strain B/Colorado/06/2017 (Fig. 1A) and drifted strain B/Brisbane/60/2008 (Fig. 1B) on pre-study (Day -3), pre-vaccination (Day 28), and 3 weeks post vaccination (Day 51). The detection limit of the assay (horizontal dashed line) was 15 PRNT50.

Figure 2. Post-challenge body weight and temperature changes

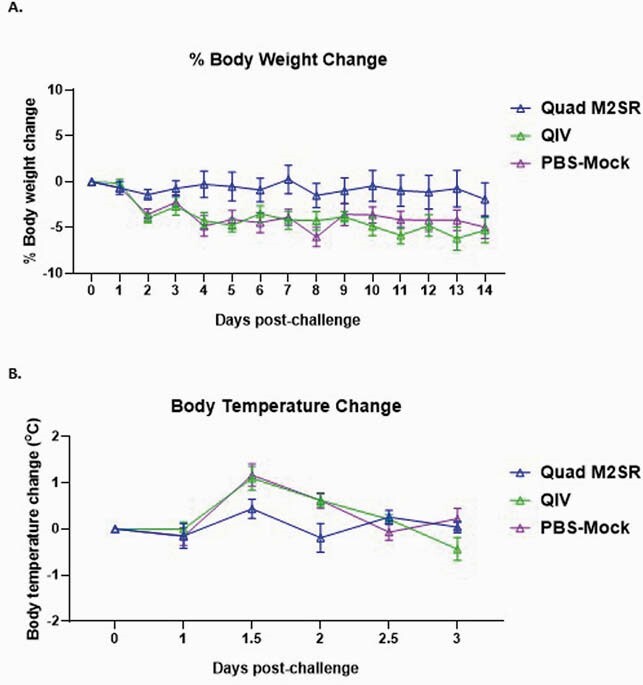

Percent body weight changes (Fig. 2A) and average body temperatures changes (Fig. 2B) following challenge with drifted Influenza B strain B/Brisbane/60/2008 for ferrets vaccinated with Quad M2SR or QIV.

Figure 3. Post-challenge virus titers in respiratory tract.

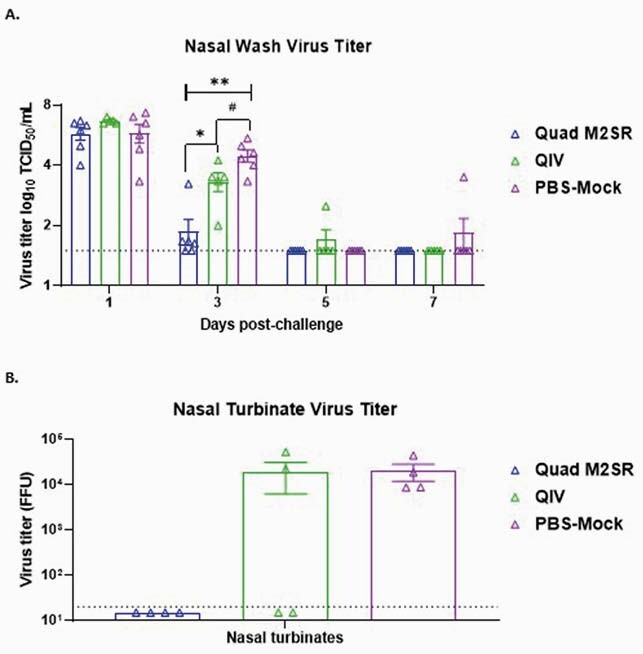

Viral titers in nasal washes (Fig. 3A) and nasal turbinates (Fig. 3B) collected post-challenge with Influenza B strain B/Brisbane/60/2008 in ferrets vaccinated with Quad M2SR or QIV. No virus was detected in the trachea or lungs. The detection limit of the assay (horizontal dashed line) was 1.5 log10 TCID50/mL and 20 FFU respectively. Virus titer between groups was significant on day 3 of the nasal washes: one-way analysis of variance (ANOVA) with Multiple t tests to compare between groups, #p<0.05,><0.01,><>

**Conclusion:**

Despite eliciting similar Ab titers, the Quad M2SR demonstrated superior protection compared to QIV in a drifted influenza B challenge model in ferrets. These results suggest that the intranasal M2SR platform may confer additional advantages over currently available vaccines. Quad M2SR is in late-stage development for testing in a first-in-human clinical study.

**Disclosures:**

**Lindsay Hill-Batorski, PhD**, **FluGen** (Employee) **Yasuko Hatta, DVM, PhD**, **FluGen** (Employee) **Michael Moser, PhD**, **FluGen** (Employee) **David Marshall, BS**, **FluGen** (Employee) **Pamuk Bilsel, PhD**, **FluGen** (Employee)

